# Classification of Covid-19 Coronavirus, Pneumonia and Healthy Lungs in CT Scans Using Q-Deformed Entropy and Deep Learning Features

**DOI:** 10.3390/e22050517

**Published:** 2020-05-01

**Authors:** Ali M. Hasan, Mohammed M. AL-Jawad, Hamid A. Jalab, Hadil Shaiba, Rabha W. Ibrahim, Ala’a R. AL-Shamasneh

**Affiliations:** 1 College of Medicine, Al-Nahrain University, Baghdad 10001, Iraq; a.hasan4@colmed-alnahrain.edu.iq; 2 College of Science, Kerbala University, Kerbala 56001, Iraq; mohammedm@uokerbala.edu.iq; 3 Faculty of Computer Science & Information Technology, University of Malaya, Kuala Lumpur 50603, Malaysia; shamasneh@siswa.um.edu.my; 4 Department of Computer Science, College of Computer and Information Sciences, Princess Nourah bint Abdulrahman University, Riyadh 84428, Saudi Arabia; HAShaiba@pnu.edu.sa; 5 Informetrics Research Group, Ton Duc Thang University, Ho Chi Minh City 758307, Vietnam; rabhaibrahim@tdtu.edu.vn; 6 Faculty of Mathematics and Statistics, Ton Duc Thang University, Ho Chi Minh City 758307, Vietnam

**Keywords:** deep learning, CT scans of lungs, fractional calculus, Q—deformed entropy, features extraction, LSTM network

## Abstract

Many health systems over the world have collapsed due to limited capacity and a dramatic increase of suspected COVID-19 cases. What has emerged is the need for finding an efficient, quick and accurate method to mitigate the overloading of radiologists’ efforts to diagnose the suspected cases. This study presents the combination of deep learning of extracted features with the Q-deformed entropy handcrafted features for discriminating between COVID-19 coronavirus, pneumonia and healthy computed tomography (CT) lung scans. In this study, pre-processing is used to reduce the effect of intensity variations between CT slices. Then histogram thresholding is used to isolate the background of the CT lung scan. Each CT lung scan undergoes a feature extraction which involves deep learning and a Q-deformed entropy algorithm. The obtained features are classified using a long short-term memory (LSTM) neural network classifier. Subsequently, combining all extracted features significantly improves the performance of the LSTM network to precisely discriminate between COVID-19, pneumonia and healthy cases. The maximum achieved accuracy for classifying the collected dataset comprising 321 patients is 99.68%.

## 1. Introduction

COVID-19 has been spreading rapidly into different countries in the world until it was classified as a pandemic by the World Health Organization (WHO). The first WHO report of confirmed cases of COVID-19 was released on 21 January 2020, with a total of 282 confirmed cases, which is comparable with the most recent report on 18 March 2020, which reached 191,127 confirmed cases. Due to this crisis, computer-aided detection/diagnosis must be employed to help radiologists in the diagnosis process to mitigate the overcapacity of a large number of COVID-19 patients. There are three common methods to diagnose COVID-19, which are blood test, X-ray, and computed tomography (CT) scan [1,2]. Among many medical imaging technologies, CT is a non-invasive technology, that has been chosen since it is regarded as a promising technique for advanced internal porosity detection and characterization [3]. According to Zonneveld [4], CT technology plays an important role in diagnostic medicine, image-guided intervention, and the assessment of therapeutic and surgical outcomes. Progress in CT technology and its applications continues to result in increasing image quality, decreasing acquisition times and dramatically expanding the clinical applications of modern CT [5]. Moreover, CT scan diagnosis is sometimes more accurate than a blood test such as CRP (C-Reactive Protein Level) [6]. As mentioned in [6], one of the cases had been tested twice negative with the CRP test while it had been diagnosed positive with a CT scan and the third test of CRP had been reported positive; which shows that CT images can be more accurate than blood tests. The CT scanner emits X-ray radiation from different angles toward the patient, and there are a set of detectors that are used to measure the density of the imaged tissue by calculating the difference between the absorbed X-rays by the patient body and the X-rays transmitted through the patient body. High-density tissue appears when the tissue absorbs the radiation to a greater amount, such as bones, while low-density tissue appears when the tissue absorbs the radiation in a lower amount, such as lungs. Therefore, discriminating boundaries between some tissues and organs or between healthy from pathological tissue within the same organ is quite challenging [5,7].

The X-ray imaging technique can be used as well as a standalone technique to diagnosis COVID-19, but because of low accuracy of the obtained results such as [8] where they get around 30% of false positive rate while [9] had 92.6% accuracy and 87.1% sensitivity which are not quite reliable enough to be considered as main diagnostic approaches.

**Figure 1:**
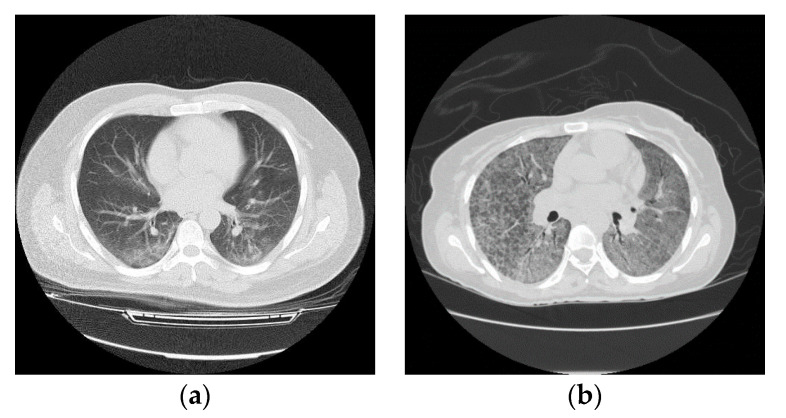
Computed tomography (CT) slices of lung in axial view: (**a**) infected lung with COVID-19, and (**b**) infected lung with pneumonia.

Although the CT is considered as an active way for initial screening and diagnosis of COVID-19, it may share certain similar texture features between COVID-19 and pneumonia, resulting in making them difficult to be differentiated as shown in Figure 1 [10].

In this study, the efficacy of convolutional neural networks (CNNs) and the proposed Q-deformed entropy are exploited for discriminating COVID-19 coronavirus, pneumonia and healthy CT lung scan. The long short-term memory (LSTM) network is considered as an extension type of artificial recurrent neural networks which is used as the classifier. The rest of the study is organized as follows: some recent previous work is demonstrated in Section 2. In Section 3, we present the proposed model. The experimentation results are presented in Section 4, and finally, Section 5 presents the conclusion and future work.

## 2. Related Work

The development of an automated system for classification of CT scans of the lung remains challenging due to the complexity of diagnosing infectious and inflammatory lung diseases in a visual examination. Although a visual examination is an acceptable standard, it is prone to errors that come from the massive number of patients that need to be diagnosed. Wang et al. [11] proposed an automated method based on using a CNN to identify the unique features of COVID-19 to provide a clinical diagnosis of the pathogenic test. The achieved accuracy of the dataset that is comprised of 217 cases, was 82.9%. Li, Qin, Xu, Yin, Wang, Kong, Bai, Lu, Fang and Song [10] proposed a fully automated framework based on using a CNN as a feature extractor to detect COVID-19 and discriminate it from pneumonia and non-pneumonia diseases. The overall accuracy to detect the COVID-19 cases of the dataset comprised of 400 CT scans, was 96%. Xu et al. [12] proposed an automated screening model based on deep learning techniques to discriminate CT cases that were infected by COVID-19 or influenza-A viral pneumonia from those cases for patients who had healthy lungs. The experimentation result revealed that the overall achieved accuracy of classifying the dataset comprised of 618 CT scans of the lung, was 86.7%. Song, et al. [13] developed an automated deep learning diagnosis system to help clinicians detect and recognize the patients who are infected by COVID-19. The collected dataset included 88, 86 and 100 CT scans of COVID-19, healthy and bacterial pneumonia cases, respectively. The proposed model is capable of classifying COVID-19 and bacterial pneumonia infected cases with an accuracy of 95%.

The existing classification models show some limitations in terms of feature extraction complexity. Feature extraction algorithms have an important role in catching the important changes in the spatial distribution of image pixels. Recently, fractional calculus and its applications were employed in different applications of sciences [14,15,16]. In this study, we develop a new handcrafted texture descriptor based on the Q-deformed entropy for image classification tasks, which is considered as one of the contributions of this study [14,15,16].

The existing models for lung CT scan classification rely on deep learning alone for feature extraction. Therefore, combining the handcrafted and deep learning features will further improve the performance of classification between COVID-19, pneumonia and healthy cases.

The motivation for this study is to propose an efficient classification of COVID-19 coronavirus, pneumonia and healthy lungs in CT scans using the combination of deep learning and Q-deformed entropy image features. Our contributions can be summarized as follows:

(1) By achieving efficient classification results under limited computational resources with the use of fewer parameters on the collected 321 chest CT scans, we have shown that the proposed approach could effectively improve the performance of classifying lungs in CT scans.
(2) The new proposed Q-deformed entropy features which are used as new texture extracted features for image classification tasks.
(3) The proposed nine layers fully convolutional network architecture which is used to extract the deep features from lungs’ CT scans.

## 3. Materials and Methods

**Figure 2:**
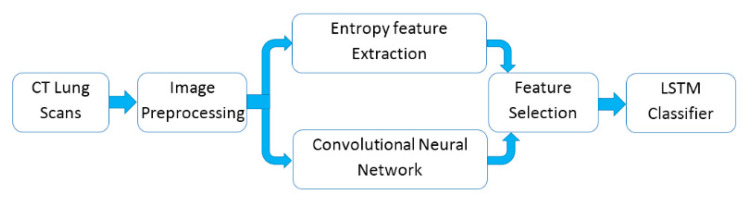
Block diagram of the proposed model. Note: Long short-term memory (LSTM).

This study aimed to improve the diagnosis process by reducing the erroneous diagnostic interpretation of CT lung scans and assist the clinicians to quickly discriminate patients who are infected by COVID-19 or pneumonia, as well as helping clinicians ignore CT lung scans of healthy patients. The proposed system comprises four main stages: CT images preprocessing, deep and handcrafted feature extraction, feature selection and finally LSTM network is used to classify these selected features into three categories (COVID-19, pneumonia and healthy) as shown in Figure 2.

### 3.1. Data Collection

A total number of 321 chest CT scans were used in this study, including 118 CT scans of infected COVID-19 patients, 96 CT scans of infected pneumonia patients and 107 CT scans of healthy people without any detectable chest infection were collected from Radiopaedia and the cancer imaging archive (TCIA) websites [17,18]. The former is an international collaborative radiology educational web resource that provides reference articles, radiology images and patients’ cases freely, and the latter includes open-access datasets for a large archive of medical images for public download. The site is sponsored by the National Cancer Institute (NCI) Cancer Imaging Program, and the University of Chicago. The healthy CT scans were collected from people who have pathologies in one lung, and another is labelled as normal.

### 3.2. CT Lung Scan Preprocessing

**Figure 3:**
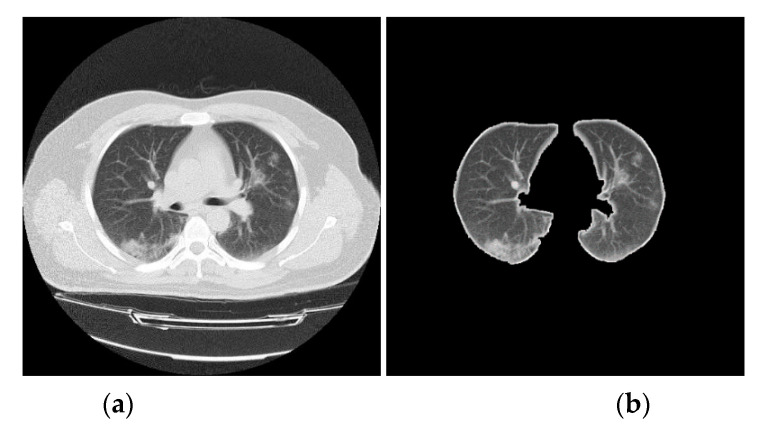
An example of lung boundary identification: (**a**) original CT slice, and (**b**) segmented CT slice.

The different types of CT artefacts that come from the image reconstruction process is based on collecting a million independent detector measurements. Any error that may occur in these measurements will affect the scan and result in intensity variations between the consecutive slices of the reconstructed CT scan [19], therefore, prior to extracting handcrafted and deep features, a set of image processes are used to normalize the intensity and reduce the effect of intensity variations between CT slices [20,21,22], and also will speed up the CNN training and improve classification performances. In addition, these processes help to identify the boundaries of a lung from its surrounding thoracic tissue in an axial view of a CT scan as it contains a high number of insignificant pixels [23]. Histogram thresholding was used to isolate the background of a CT lung scan by thresholding the intensity values by the mean value of each CT slice individually. Subsequently, a set of morphological operations such as dilation and hole filling are implemented to remove any hole appearing in the segmented image [23,24,25]. Then the deficiencies of the segmentation process are overcome by removing all small connected objects. Consequently, a binary mask with ones representing the lung, and zeros representing the background, is multiplied with the original CT lung scan to extract only the effective pulmonary regions [12]. The pseudo-code for the preprocessing of CT lung scan steps is shown in Algorithm 1. Figure 3 displays a sample of how a CT lung slice is segmented.
**Algorithm 1**: Pseudo-code for CT lung scans preprocessing.  Input: Input image I(n,m) Output: Output image K(n,m) begin Adjust image intensity values Convert the image into a binary image(B)   For all I pixels:     IF the grayscale value < the image Mean,      THEN, the pixel value = 0     ELSE the pixel grayscale value = 255     End IF  End For Remove small objects from binary image, and Fill image regions and holes Produce the output image(K) For each Input image I do      For i = 1 to n do       For j = 1 to m do        Multiply each element in I(i,j) by the corresponding element         in B(i,j) and return the output image(K)      End For     End For End For

### 3.3. Q-Deformed Entropy Feature Extraction (QDE)

Texture feature is an important aspect in many medical image analyses which provides a significant advantage in medical image classification. The concept of entropy has been generalized and applied in many scientific disciplines [26]. The entropy-based algorithms are the most significant feature extraction methods which are capable of detecting the image’s small changes in intensity values, as well as sharpening the texture details [27].

Inspired by the deformation theory in q-calculus, which has been explored intensively in several physical disciplines, we propose the QDE feature extraction model for extracting the texture features from CT scans.

Deformation theory is the investigation of accurate settings which are connected with changeable solutions. The accurate settings are, therefore, the consequence of employing the method of fractional differential calculus to resolve a problem with restraints. Quantum calculus, named calculus without limits at times, corresponds to traditional accurate calculus, without the concept of limits. Quantum calculus defines “q-calculus” as shown in Equation (1):

(1) $$\Delta_{q}I\left(x\right)=\frac{I\left(qx\right)-I\left(X\right)}{\left(q-1\right)x},$$

where *I* is a function and *x* indicates the variable.

The q-logarithm deformation is given by Equation (2) [28]:

(2) $$ln_{q}\;\left(x\right)=\{\begin{array}{cc} \ln\left(x\right) & q=1,x>0\\ \frac{x^{1-q}-1}{1-q} & q\neq 1,\;x>0\\ Undefined & x\leq 0 \end{array}.$$

The Box–Muller transform is a process used to modify many recent concepts such as entropy and probability density functions. Equation (2) implies the generalized q-deform Box–Muller transform as shown in Equation (3):

(3) $$BM_{q}\;\left(x\right)=\sqrt{-2ln_{\hat{q}}\;\left(x\right)\;}=\sqrt{\left(-2\right)\left(\frac{x^{1-\hat{q}}-1}{1-\hat{q}}\right)\;}=\sqrt{\left(-2\right)\left(\frac{x^{1-\left(\frac{1+q}{3-q}\right)}-1}{1-\left(\frac{1+q}{3-q}\right)}\right)},$$

where $\hat{q}=\frac{1+q}{3-q},\;q\neq 3$.

To apply the QDE for image texture feature extraction, we will consider the value of the pixel as a positive number, therefore, to avoid complex numbers, we use the radius (|q|). Hence, by solving Equation (3) for |*q*|, taking into account the positive value of *x*, we have |*q*|< 3.

The suggested technique computes the above $\hat{q}$-BM based on occurrence information of the effort image, which provides a consistency asset to investigate its construction. The improvement of the $\hat{q}$-BM fractional function is that it is sensitive to non-textured sections (with low occurrence). Moreover, it improves any variations in texture specifics in the sections, where pixel standards are shifting sharply (high occurrence).

We proceed to define the QDE by applying Equation (4). Entropy is designed in two behaviours; the first is the entropy transformation (ΔE) to a scheme (image) covering a sub-scheme. The second computes the absolute entropy (E) of the image based on its individual pixels. Roughly, it gives the probability of the image’s organism in that state. In this situation, it efficiently describes entropy independently from its properties due to changes, which may involve energies. In addition, it contains logical situations such as information of the image (see [29]). Following the above treatment, we utilize the entropy transformation (ΔE) as follows in Equation (4):

(4) $${\left(\Delta E\right)}_{q}\left(x\right)=\frac{BM_{q}\;\left(p\left(x\right)\right)}{L}=\frac{\sqrt{\left(-2\right)\left(\frac{p_{i}{\left(x\right)}^{1-\left(\frac{1+q}{3-q}\right)}-1}{1-\left(\frac{1+q}{3-q}\right)}\right)\;}}{\sum_{i=1}^{n}{\left(p_{i}\left(x\right)\right)}^{2}},$$

where 0 < *q* < 3, *L* indicates the localized change of the image (*L* is usually referred to as an energy of the image, which represents the difference between the lightness and the darkness in the grey images and the difference in colours in coloured images in the suggested domain). The formula of *L* was determined by the probability of the pixel as well as the *BMq*. Equation (4) designates that the transformation is flexible because it displays a comparative association between entropy and the energy movement, in the image.

The proposed QDE depends on the image detail, and on the grey level’s intensity which is characterized by the texture property of the image.

For our proposed QDE texture extraction, we first divide the CT lung image into non-overlapping blocks with size m x m pixels, then the QDE for each block is computed. In total, 16 QDE features are extracted from each CT lung image. The pseudo-code for the proposed QDE is described in Algorithm 2.
**Algorithm 2**: Pseudo-code for the proposed Q-deformed entropy feature extraction (QDE) algorithm.  Initialization: I = Input image, 0 < q < 3 For each Input image I do     (b1, b2, …, bn) ← divide I into n blocks of size m x m pixels    For i = 1 to n do        QDE in Equation (5), where i denotes the ith block of m x m        dimension     End For    QDE ← I = (1, 2, …n) // QDE Features of all (n) blocks End For

### 3.4. Deep Learning for Feature Extraction

The deep learning method has been proven to be suitable as a feature extractor in many computer vision systems that can be used to enhance the classification accuracies. Although the handcrafted feature extraction method has been verified to be sufficient for classification tasks, their performances based on expert knowledge reflect limited aspects of the problem. To extract more efficient features, we further use the CNN method to learn a feature extraction model which has proven to be a powerful method in many computer vision systems. By combining the deep learning and handcrafted image features, the classification accuracy has been enhanced.

The convolutional neural network (CNN) is an adapted version of the traditional neural network architecture, comprised of three layers; convolutional, pooling and fully-connected layers [25,30]. Each convolutional layer includes a set of kernels or also named convolutional filters (Cf), that are responsible for determining a tensor of feature maps; these kernels convolve the input volume entirely by moving with a certain amount named “stride(s)” which is chosen in such a way that makes the output volume dimensions as integers [25,31]. Due to the striding process, the spatial dimensions of the input volume shrink dramatically after each convolutional layer. Therefore, to preserve the original input volume and low level of features, zero-padding is needed to pad the input volume with zeros [25,32]. Then, all negative numbers in the feature maps are set to zero through the rectified linear unit (ReLU) layer, which is used to increase the nonlinearity of the feature maps. Subsequently, pooling layers are used to reduce the dimensionality by partitioning the feature maps into small non-overlapped regions [25,33]. In this study, the max pooling function is used for dimensionality reduction in the pooling layer of CNN. Thereafter, the batch normalization layer is used to accelerate the training process and regulate the CNN, by normalizing the feature maps. Finally, the normalized feature maps are fed to the fully connected layer (FC) which represents the most important layer in the CNN. The fully connected layer is used as a classifier to derive the final decision and to give the final probability for each label [25,30,31].

The CNN has verified to be a powerful approach for feature extraction, but it still has the over-fitting problem, due to a huge number of the network’s parameters that need to be trained. Therefore, we used a few convolution layers in order to minimize the over-fitting by reducing the CNN architecture complexity. 

The main objective of the CNN is to extract the high-level features for a specific task. Therefore, it is necessary to know how the network is architected, the number and size of convolutional layers, how these layers are connected and how the CT slices are fed to the network. In this study, to avoid the computation complexity of CNN, the dimensions of CT slices are resized to 256 × 256 pixels. The size of convolutional layers and the number of zero-padding (ZP) are determined for a given CT slice by using Equations (5)–(8), as shown in the following [25,34,35]:

(5) $$Conv_{width}=\frac{CTSlice_{width}-Cf_{width}+\left(2\times ZP_{width}\right)}{S_{width}}+1,\;$$

(6) $$Conv_{height}=\frac{CTSlice_{height}-Cf_{height}+\left(2\times ZP_{height}\right)}{S_{height}}+1,\;$$

(7) $$ZP_{width}=\frac{Cf_{width}-1}{2},\;\mathrm{and}\;\;\;\;\;\;\;\;$$

(8) $$ZP_{height}=\frac{Cf_{height}-1}{2}.$$

**Figure 4:**
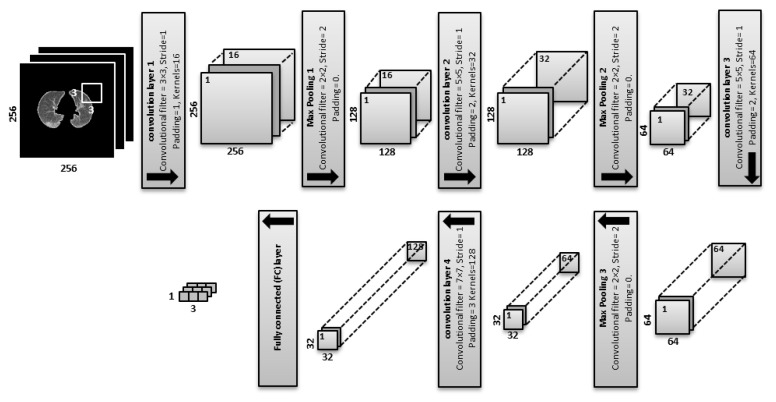
Architecture of the convolutional neural network (CNN) as a feature extractor with four convolutional layers, three pooling layers and one fully connected layer.

The proposed CNN architecture with a given input CT slice of 256 × 256 pixels is shown in Figure 4:

- $Conv_{1}$ (filters of size 3 × 3, stride of 1, padding of 1, and kernels of 16) are applied:
  $$Conv_{width,height}=\frac{256-3+\left(2\times 1\right)}{1}+1=256.$$
  For the feature maps, we have 256 × 256 × 16 = 1,048,576 neurons.
- $Max\;Pooling_{1}$ is equal to the previous feature maps divided by the stride number:
  $$Max\;Pooling_{1}=\frac{256}{2}=128.$$
  For the feature maps, we have 128 × 128 × 16 = 262,144 neurons in the feature map of the first max pooling layer.
- $Conv_{2}$ (filters of size 5 × 5, a stride of 1, padding of 2 and kernels of 32) are applied:
  $$Conv_{width,height}=\frac{128-5+\left(2\times 2\right)}{1}+1=128.$$
  For the feature maps, there are 128 × 128 × 32 = 524,288 neurons.
- $Max\;Pooling_{2}$ is equal to the previous feature maps divided by the stride number:
  $$Max\;Pooling_{2}=\frac{128}{2}=64.$$
  For the feature maps, we have 64 × 64 × 32 = 131,072 neurons.
- $Conv_{3}$ (filters of size 5 × 5, a stride of 1, padding of 2 and kernels of 64) are applied:
  $$Conv_{width,height}=\frac{64-5+\left(2\times 2\right)}{1}+1=64.$$
  For the feature maps, we have 64 × 64 × 64 = 262,144 neurons in the feature map of the third convolution layer.
- $Max\;Pooling_{3}$ is equal to the previous feature maps divided by the stride number:
  $$Max\;Pooling_{3}=\frac{64}{2}=32.$$
  For the feature maps, we have 32 × 32 × 64 = 65,536 neurons.
- $Conv_{4}$ (convolutional filters of size 7 × 7, a stride of 1, padding of 3 and kernels of 128) are applied:
  $$Conv_{width,height}=\frac{32-7+\left(2\times 3\right)}{1}+1=32.$$
  For the feature maps, we have 32 × 32 × 128 = 131,072 neurons.
- The fully connected (FC) layer determines the class scores by combining all features which are produced and learned by the previous layers to produce a feature map of size 1 × 1 × 3, that is equal to the number of classes in the dataset. The input size of the FC layer is equal to 131,072 that is produced by $Conv_{4}$.

Then the QDE features and deep features are combined and refined by employing the analysis of variance (ANOVA) method. ANOVA is an efficient statistical method, used for eliminating and ignoring the irrelevant and redundant features in the feature vector. It assesses features by determining both an F-statistic value and a *p*-value. The F-statistic is a ratio of between-class variance to within-class variance, while the P-value is the probability of the test statistic being at least equal to or less than the critical value of the test (5% or 1%). ANOVA was explained in details in [36].

### 3.5. LSTM Neural Network Classifier

**Figure 5:**
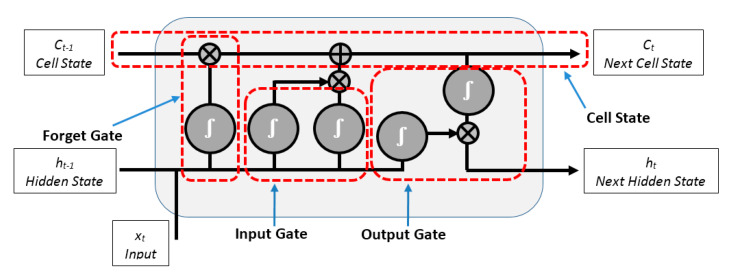
The structure of Long short-term memory (LSTM).

LSTM is a powerful artificial neural network, proposed by Hochreiter and Schmidhuber in the middle of the 1990s to address the substantial limitation of artificial neural networks when dealing with sequences of data. It is a type of recurrent neural network (RNN) which can learn and remember input data through the use of gates. These gates are used to regulate the information within the network by discarding information from previous steps that results in the loss of important information in the next steps [37,38]. The LSTM is composed of a cell state, input and forget and output gates as shown in Figure 5, where, *x_t_* is the current input, *C_t_* and *C*_*t*−1_ denote the new updated cell state and cell state from last LSTM unit, respectively, *h_t_* and *h*_*t*−1_ represent the current output and the output of the last LSTM unit, respectively [37]. The forget gate uses the previous LSTM’s output *h*_*t*−1_ and the current input *x_t_* to produce a vector of numbers, ranging from 0 to 1, corresponding to each value in *C*_*t*−1_ to decide which information should be kept and which should be discarded from *C*_*t*−1_. The forget gate is given by [39]:

(9) $$f_{t}=\sigma \left(W_{f}\left[h_{t-1},x_{t}\right]+b_{f}\right),\;\;$$

where, *σ* denotes the sigmoid function, *W_f_* and *b_f_* are the weighted matrices, and the bias of the forget gate of LSTM, respectively.

While the input gate of LSTM uses the sigmoid function and tanh function respectively to decide which values in the current input *x_t_* and the output of the previous LSTM *h*_*t*−1_ are important and allow them to pass to the next gate after normalizing them into a new range between −1 and 1 to regulate the LSTM network by using Equations (10)–(12), as shown in the following [40]:

(10) $$M_{t}=\sigma \left(W_{i}\left[h_{t-1},x_{t}\right]+b_{i}\right),\;$$

(11) $$N_{t}=tanh\left(W_{i}\left[h_{t-1},x_{t}\right]+b_{i}\right)\;\mathrm{and}\;\;$$

(12) $$i_{t}=N_{t}\;M_{t},$$

where, *M_t_* and *N_t_* are the outputs of the sigmoid function and the tanh function, respectively. *W_i_* and bi are the weighted matrices and the bias of the input gate of LSTM, respectively. Then, the previous cell state *C*_*t*−1_ is updated by multiplying it with the forget gate output to drop values in the cell state if the corresponding values in the forget gate output are close to 0 as given in Equation (13) [32,37].

(13) $$C_{t}=C_{t-1}\;f_{t},$$

where, *C_t_* is the current cell state of LSTM network.

Then, the current cell *C_t_* is updated by adding the output of the input gate, as given in Equation (14) [22]:

(14) $$C_{t}=C_{t}+i_{t}.$$

Finally, the output gate determines the new output values based on multiplying the new updated cell state *C_t_* after passing through a tanh function and the previous output *h*_*t*−1_ after passing through a sigmoid function, as given in Equation (15) and Equation (16):

(15) $$O_{t}=\sigma \left(W_{o}\left[h_{t-1},x_{t}\right]+b_{o}\right),$$

(16) $$h_{t}=O_{t}\tanh\left(C_{t}\right),\;\;$$

where, $W_{o}$ and $b_{o}$ are the weighted matrices and the bias of the output gate of LSTM, respectively.

Consequently, the information that is relevant to be kept from the previous LSTMs units is decided by the forget gate. The information that is relevant to be added from the current LSTM network is decided by the input gate. The next output is determined by the output gate.

The LSTM was improved by the bidirectional LSTM network (BiLSTM), where, it trains two LSTM layers instead of one; one works in a forward direction of the input sequence and the second works in a backward direction of the input sequence. This can improve and accelerate the network performance in classifying sequential data [32,37].

In this study, the LSTM network contains seven layers; sequence input with 14 dimensions that come from combining the QDE and DF features.

## 4. Experimental Results

Experimentally, there are 16 features, extracted from each CT lung image, coming from combining the features that are extracted by QDE and CNN methods. Definitely not all the extracted features are significant, and high dimensionality of a feature vector can negatively affect the classification accuracy [41]. ANOVA is applied to measure t the extracted features, where the number of features is reduced from 19 to 14 significant predictors.

**Figure 6:**
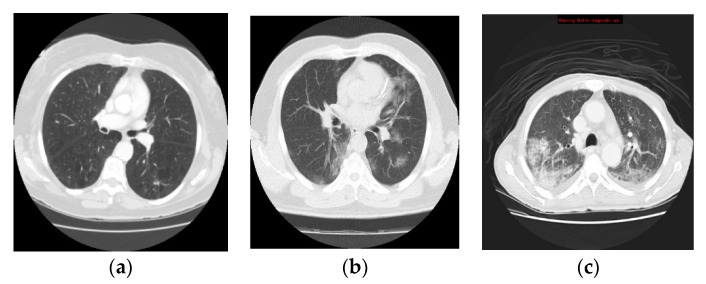
CT scans of the lung from the collected dataset: (**a**) healthy lung, (**b**) COVID-19 infection and (**c**) pneumonia infection.

Contrast-enhancement CT scan axial viewing is preferred for the clinicians to diagnose lung disease. Therefore, it is used in this study due to being highly sensitive to COVID-19 and pneumonia infections. The collected dataset is adopted to evaluate the proposed method. During the training scenario of CNN and LSTM networks, 70% of CT scans are used for the training phase, and the remaining 30% of the CT scans are used to assess the final classification performance. Figure 6 shows a sample of the CT lung images of normal, COVID-19 and pneumonia infected patients from the collected dataset.

The optimum number of convolutional layers, neurons, pooling layers, learning rate and a kernel size of CNN were determined experimentally. The proposed CNN consists of nine layers as summarized in Table 1, and trained with the following parameters that are set experimentally: the momentums are 0.9 with a learning rate of 0.0001, the maximum number of epochs is 20 with the minimum batch size of 128, and the maximum iteration number is 500. The code was developed using MATLAB 2019b.

**Figure 7:**
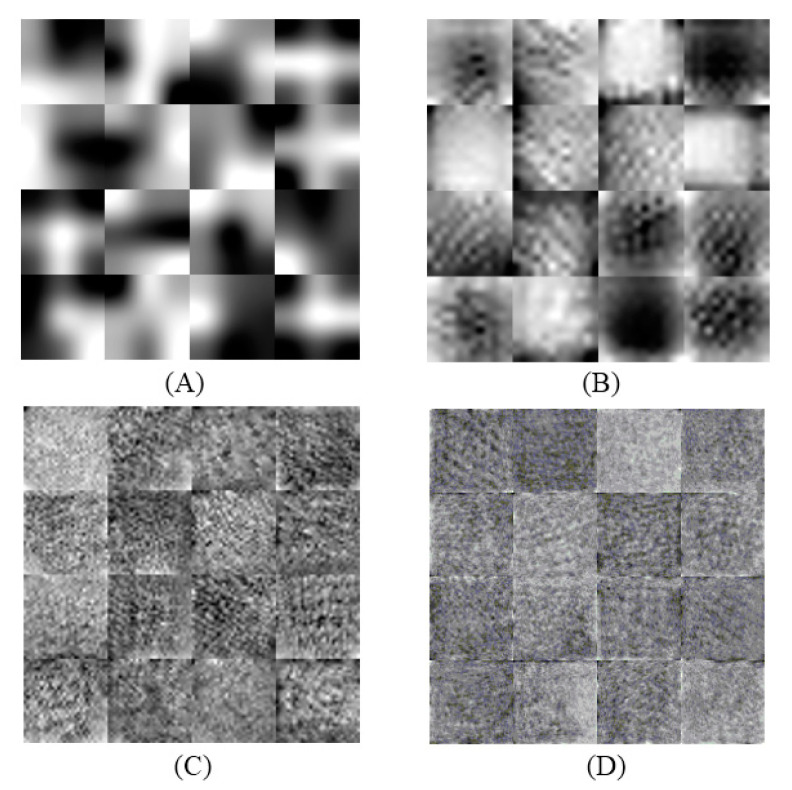
The images of learned weights of the CNN layers: (**A**) Conv1 (1 × 16), (**B**) Conv2 (1 × 32), (**C**) Conv3 (1 × 46), and (**D**) Conv4 (1 × 128).

**Figure 8:**
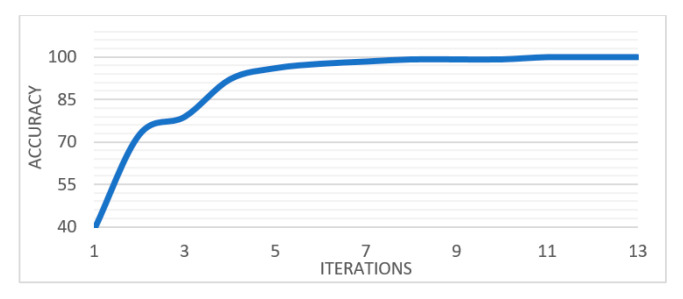
The training process of the proposed CNN.

Figure 7 shows the weights learned at the four convolutional layers of CNN in an image form. Figure 8 shows how the training process with the number of iterations. This denotes that the proposed architecture of CNN has a good performance in extracting deep features (DF) from CT scans of lungs. The performance of the combined features (QDE–DF) is evaluated by comparing the true positive (TP) values of the three groups with the performance of each method (QDE and DF) when using them individually as demonstrated in Table 2.

The first row of Table 2 shows that the lowest classification accuracy result was 97.50%, which was obtained by using the QDE method (handcrafted) image features which proves that the proposed QDE has efficiently encoded the CT scan texture information. The second row of Table 2 illustrates 98% classification accuracy using the DF image features. However, using the combination of QDE method (handcrafted) and deep features (DF) has further increased the classification accuracy to up to 99.68%. As indicated by these experimentation results, the classification accuracy produced by the combination of QDE–DF is slightly higher than that produced without the features’ combination. However, the combination of feature vectors has increased the dimensionality of the final image features.

In detail, we extract a 14-component feature vector by using the QDE method and a 3-component feature vector by using the CNN method. As a result, the final feature vector is a vector with a 17-dimensional space (14 + 3).

Additionally, the performance of the LSTM classifier is compared with the achieved accuracy of other classifiers such as linear SVM, KNN and logistic regression, as demonstrated in Table 3. These results prove the superiority of LSTM network to classify MRI brain scans precisely.

The best accuracy of 99.68% is achieved by the combined QDE–DF features where the LSTM succeeds to classify all CT scans of infected patients with COVID-19 as well as healthy patients correctly with TP of 100% while only one infected patient with pneumonia failed to be classified by LSTM model with TP of 98.9%.

**Figure 9:**
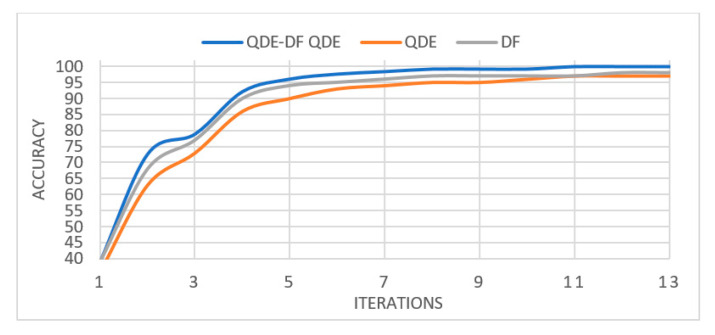
The training process of LSTM network with DF, LBP and LBP-DF features.

Figure 9 shows that the performance of LSTM with the combined features QDE–DF outperforms its performance when using QDE and DF features individually.

In this study, the combined feature set QDE–DF and the robustness of the LSTM network increased the classification efficiency of CT scans of lung significantly.

## 5. Conclusions

In this study, we proposed a feature extraction method based on QDE–DF to improve the classification process for discriminating COVID-19 coronavirus, pneumonia and healthy CT lung scans. This feature set is based mainly on the proposed CNN architecture that is used to extract the spectral and spatial deep features of the scan. The best design of the CNN network that is used to extract DF depends essentially on how to choose parameters of the CNN network where the optimal parameters have an immediate effect on the classification accuracy and computational complexity. The parameters of CNN such as the number of convolutional layers, convolution kernel size, padding, stride and number of neurons, are chosen experimentally; nine layers with (3 × 3), (5 × 5), (5 × 5) and (7 × 7) convolution kernel sizes respectively and a (2 × 2) pooling kernel in each layer. One interesting direction for future work is to apply the proposed model on another CT scan modality. In summary, this study illustrates the usability of CNN as a feature extractor and of the LSTM network as a classifier to attain an accuracy of 99.68% of classifying CT scans of the lung.

## Figures and Tables

**Table 1:** The proposed model of CNN (convolutional neural network).

Layer Name	Kernel Size	Feature Map
Input layer	(256 × 256)	
Conv1	(3 × 3)	(256 × 256 × 16)
Max. Pooling1	(2 × 2)	(128 × 128 × 16)
Conv2	(5 × 5)	(128 × 128 × 32)
Max. Pooling2	(2 × 2)	(64 × 64 × 32)
Conv3	(5 × 5)	(64 × 64 × 64)
Max. Pooling3	(2 × 2)	(32 × 32 × 64)
Conv4	(7 × 7)	(32 × 32 × 128)
FC	(1 × 3)	(1 × 3)

**Table 2:** Comparisons of QDE (Q-deformed entropy), deep features (DF) and the proposed QDE–DF using LSTM (long short-term memory).

Method	Accuracy 100%	TP 100% COVID-19	TP 100% Healthy	TP 100% Pneumonia
QDE	97.50	95.70	100	96.80
DF	98	97.40	100	96.80
QDE–DF	99.68	100	100	98.90

**Table 3:** Classification results obtained from SVM, KNN, logistic regression and LSTM.

Method	Accuracy 100%	TP 100% COVID-19	TP 100% Healthy	TP 100% Pneumonia
Linear SVM	96.20	94.90	98.10	95.80
KNN	95.30	93.20	97.20	95.80
Logistic Regression	97.20	96.60	98.10	96.80
LSTM	99.68	100	100	98.90

## References

[1] Fan L., Li D., Xue H., Zhang L., Liu Z., Zhang B., Zhang L., Yang W., Xie B., Duan X. (2020). Progress and prospect on imaging diagnosis of COVID-19. Chin. J. Acad. Radiol..

[2] Hu Z., Song C., Xu C., Jin G., Chen Y., Xu X., Ma H., Chen W., Lin Y., Zheng Y. (2020). Clinical Characteristics of 24 Asymptomatic Infections with COVID-19 Screened among Close Contacts in Nanjing, China.

[3] Hermanek P., Carmignato S. (2017). Porosity measurements by X-ray computed tomography: Accuracy evaluation using a calibrated object. Precis. Eng..

[4] Zonneveld F.W., Hanafee W.N. (1988). Computed Tomography of the Temporal Bone and Orbit. J. Comput. Assist. Tomogr..

[5] Samei E. (2020). Computed Tomography: Approaches, Applications, and Operations.

[6] Abdullayev C.-P. COVID-19 Pneumonia. https://radiopaedia.org/cases/covid-19-pneumonia-45?lang=us.

[7] Depeursinge A., Al-Kadi O.S., Mitchell J.R. (2017). Biomedical Texture Analysis: Fundamentals, Tools and Challenges.

[8] Zhang J., Xie Y., Li Y., Shen C., Xia Y. (2020). COVID-19 Screening on Chest X-ray Images Using Deep Learning based Anomaly Detection. arXiv.

[9] Wang L., Wong A. (2020). COVID-Net: A tailored deep convolutional neural network design for detection of COVID-19 cases from chest radiography images. arXiv.

[10] Li L., Qin L., Xu Z., Yin Y., Wang X., Kong B., Bai J., Lu Y., Fang Z., Song Q. (2020). Artificial Intelligence Distinguishes COVID-19 from Community Acquired Pneumonia on Chest CT. Radiology.

[11] Wang S., Kang B., Ma J., Zeng X., Xiao M., Guo J., Cai M., Yang J., Li Y., Meng X. (2020). A deep learning algorithm using CT images to screen for Corona Virus Disease (COVID-19). MedRxiv.

[12] Xu X., Jiang X., Ma C., Du P., Li X., Lv S., Yu L., Chen Y., Su J., Lang G. (2020). Deep Learning System to Screen Coronavirus Disease 2019 Pneumonia. arXiv.

[13] Song Y., Zheng S., Li L., Zhang X., Zhang X., Huang Z., Chen J., Zhao H., Jie Y., Wang R. (2020). Deep learning Enables Accurate Diagnosis of Novel Coronavirus (COVID-19) with CT images. MedRxiv.

[14] Yang X.-J., Gao F., Ju Y. (2020). General Fractional Derivatives with Applications in Viscoelasticity.

[15] Yang X.-J. (2019). General Fractional Derivatives: Theory, Methods and Applications.

[16] Al-Shamasneh A.R., Jalab H.A., Shivakumara P., Ibrahim R.W., Obaidellah U.H. (2020). Kidney segmentation in MR images using active contour model driven by fractional-based energy minimization. SignalImage Video Process..

[17] Radiopaedia (2020). COVID-19 CT Cases. www.radiopaedia.org.

[18] Archive C.I. (2019). SPIE-AAPM-NCI Lung Nodule Classification Challenge Dataset. www.cancerimagingarchive.net.

[19] Barrett J.F., Keat N. (2004). Artifacts in CT: Recognition and avoidance. Radiographics.

[20] Tantisatirapong S. (2015). Texture Analysis of Multimodal Magnetic Resonance Images in Support of Diagnostic Classification of Childhood Brain Tumours.

[21] Nabizadeh N., Kubat M. (2015). Brain tumors detection and segmentation in MR images: Gabor wavelet vs. statistical features. Comput. Electr. Eng..

[22] Ibrahim R.W., Hasan A., Jalab H.A. (2018). A new deformable model based on fractional Wright energy function for tumor segmentation of volumetric brain MRI scans. Comput. Methods Programs Biomed..

[23] Mansoor A., Bagci U., Foster B., Xu Z., Papadakis G.Z., Folio L.R., Udupa J.K., Mollura D.J. (2015). Segmentation and image analysis of abnormal lungs at CT: Current approaches, challenges, and future trends. RadioGraphics.

[24] Gonzalez R., Woods R. (2002). Digital Image Processing.

[25] Hasan A.M., Jalab H.A., Meziane F., Kahtan H., Al-Ahmad A.S. (2019). Combining deep and handcrafted image features for MRI brain scan classification. IEEE Access.

[26] Jalab H.A., Subramaniam T., Ibrahim R.W., Kahtan H., Noor N.F.M. (2019). New Texture Descriptor Based on Modified Fractional Entropy for Digital Image Splicing Forgery Detection. Entropy.

[27] Al-Shamasneh A.a.R., Jalab H.A., Palaiahnakote S., Obaidellah U.H., Ibrahim R.W., El-Melegy M.T. (2018). A new local fractional entropy-based model for kidney MRI image enhancement. Entropy.

[28] Umarov S., Tsallis C., Steinberg S. (2008). On a q-central limit theorem consistent with nonextensive statistical mechanics. Milan J. Math..

[29] Callen H.B. (1998). Thermodynamics and an Introduction to Thermostatistics.

[30] Chang P., Grinband J., Weinberg B., Bardis M., Khy M., Cadena G., Su M.-Y., Cha S., Filippi C., Bota D. (2018). Deep-learning convolutional neural networks accurately classify genetic mutations in gliomas. Am. J. Neuroradiol..

[31] Gu J., Wang Z., Kuen J., Ma L., Shahroudy A., Shuai B., Liu T., Wang X., Wang G., Cai J. (2018). Recent advances in convolutional neural networks. Pattern Recognit..

[32] Kutlu H., Avcı E. (2019). A Novel Method for Classifying Liver and Brain Tumors Using Convolutional Neural Networks, Discrete Wavelet Transform and Long Short-Term Memory Networks. Sensors.

[33] Lundervold A.S., Lundervold A. (2019). An overview of deep learning in medical imaging focusing on MRI. Zeitschrift für Medizinische Physik.

[34] Alom M.Z., Taha T.M., Yakopcic C., Westberg S., Sidike P., Nasrin M.S., Hasan M., Van Essen B.C., Awwal A.A., Asari V.K. (2019). A State-of-the-Art Survey on Deep Learning Theory and Architectures. Electronics.

[35] Duan M., Li K., Yang C., Li K. (2018). A hybrid deep learning CNN–ELM for age and gender classification. Neurocomputing.

[36] Dubitzky W., Granzow M., Berrar D.P. (2007). Fundamentals of Data Mining in Genomics and Proteomics.

[37] Le X.-H., Ho H.V., Lee G., Jung S. (2019). Application of long short-term memory (LSTM) neural network for flood forecasting. Water.

[38] Chen G. (2016). A gentle tutorial of recurrent neural network with error backpropagation. arXiv.

[39] Tsiouris Κ.Μ., Pezoulas V.C., Zervakis M., Konitsiotis S., Koutsouris D.D., Fotiadis D.I. (2018). A Long Short-Term Memory deep learning network for the prediction of epileptic seizures using EEG signals. Comput. Biol. Med..

[40] Sak H., Senior A., Beaufays F. Long Short-Term Memory Recurrent Neural Network Architectures for Large Scale Acoustic Modeling. Proceedings of the Fifteenth Annual Conference of the International Speech Communication Association.

[41] Babatunde O., Armstrong L., Leng J., Diepeveen D. (2014). A genetic algorithm-based feature selection. Br. J. Math. Comput. Sci..

